# Spindle cell hemangioma of the lower abdominal wall mimicking dermatofibrosarcoma protuberans: A case report

**DOI:** 10.1016/j.jdcr.2026.06.025

**Published:** 2026-06-18

**Authors:** Hiroki Nagasawa, Hiroaki Kuwahara, Keigo Ito, Rei Ogawa

**Affiliations:** a Department of Plastic and Reconstructive Surgery, Nippon Medical School Musashi Kosugi Hospital, Kawasaki, Japan; b Department of Dermatology, Nippon Medical School Musashi Kosugi Hospital, Kawasaki, Japan; c Department of Plastic, Reconstructive and Cosmetic Surgery, Nippon Medical School Hospital, Tokyo, Japan

**Keywords:** abdominal wall, CD34, dermatofibrosarcoma protuberans, spindle cell hemangioma, vascular tumor

## Introduction

*Spindle cell hemangioma* (SCH) is a rare benign vascular neoplasm arising in the skin and subcutaneous tissue, predominantly affecting young to middle-aged adults. It most commonly develops in the distal extremities, particularly the fingers and feet, and occurrence in the trunk is considered extremely rare. SCH typically presents as a small subcutaneous nodule; cases manifesting as a relatively large mass in the trunk have rarely been reported. Therefore, when a relatively large spindle cell tumor arises in the trunk, differentiation from mesenchymal tumors such as *dermatofibrosarcoma protuberans* (DFSP) may be challenging based on clinical presentation and biopsy findings alone.

Here we report an extremely rare case of SCH arising in the trunk, in which differentiation from DFSP was required at the time of diagnosis, with a brief review of the literature.

## Case report

The patient was a man in his 20s who first noticed a mass in the lower abdomen in early 2024. Several months later, the mass became tender. He visited a local dermatologist in June 2024 and was observed under a presumptive diagnosis of a benign subcutaneous tumor. Computed tomography at that time revealed a well-defined soft tissue mass approximately 3 cm in diameter in the lower abdominal subcutaneous tissue, without deep fascial invasion or calcification. The mass gradually enlarged over approximately 1 year, after which he was referred to our department.

At the initial visit to our department, a firm subcutaneous mass of approximately 5 cm was palpable in the lower abdomen with tenderness. There was no overlying skin erythema or ulceration, and no inguinal lymphadenopathy. Magnetic resonance imaging revealed a multilocular mass of up to approximately 5 cm projecting from the lower abdominal subcutaneous tissue toward the skin surface, demonstrating isointense to mildly hyperintense signal on T1-weighted images and heterogeneously hyperintense signal on T2-weighted images, with internal findings suggestive of hemorrhage and thrombosis. Although no fascial or muscular invasion was identified, mesenchymal tumors including DFSP were included in the differential diagnosis. Ultrasound demonstrated a well-defined mass with abundant internal blood flow signals, suggesting a hypervascular lesion.

Core needle biopsy was performed for definitive diagnosis. Histopathological examination revealed proliferation of spindle cells with mild atypia, and immunohistochemical staining demonstrated CD34 positivity, raising suspicion for DFSP. As malignancy could not be completely excluded, complete tumor excision was performed under general anesthesia. The tumor was located within the subcutaneous tissue with relatively well-defined margins, and regions with abundant vascular structures were noted intraoperatively. Intraoperative blood loss was minimal, and the tumor was resected en bloc (Fig 1).

**Fig 1:**
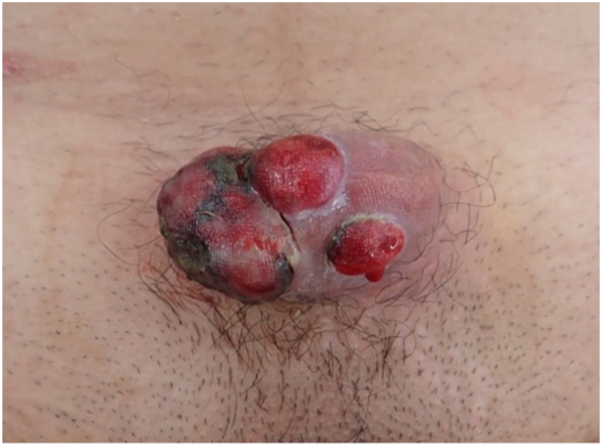
Preoperative photograph showing a tumor measuring 5 cm in the widest diameter in the lower abdominal midline.

The postoperative course was uneventful, and the patient was discharged on postoperative day 5. Histopathological examination of the excised specimen revealed multinodular proliferation of spindle cells with dilated vascular lumen formation and intraluminal thrombosis (Fig 2). Immunohistochemical staining demonstrated CD34 and CD31 positivity restricted to vascular endothelial cells, with limited expression of these markers in the spindle cell component (Fig 3). S-100, α-smooth muscle actin, desmin, D2-40, and HHV8 were negative; Ki-67 was low (1% to 3%). Cellular atypia was mild and mitotic figures were scarce. Based on these findings, a final diagnosis of SCH was established. At 6-month follow-up, the patient remains free of recurrence.

**Fig 2:**
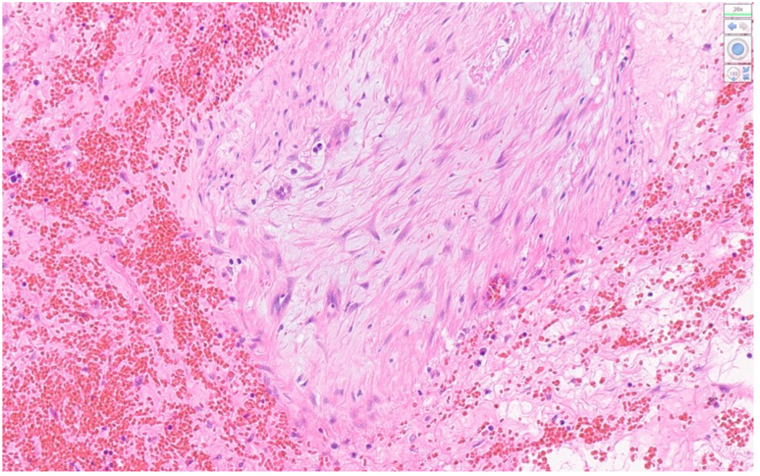
Hematoxylin and eosin staining of the excised specimen. Spindle cells are observed centrally, with abundant erythrocytes in the surrounding dilated vascular spaces.

**Fig 3:**
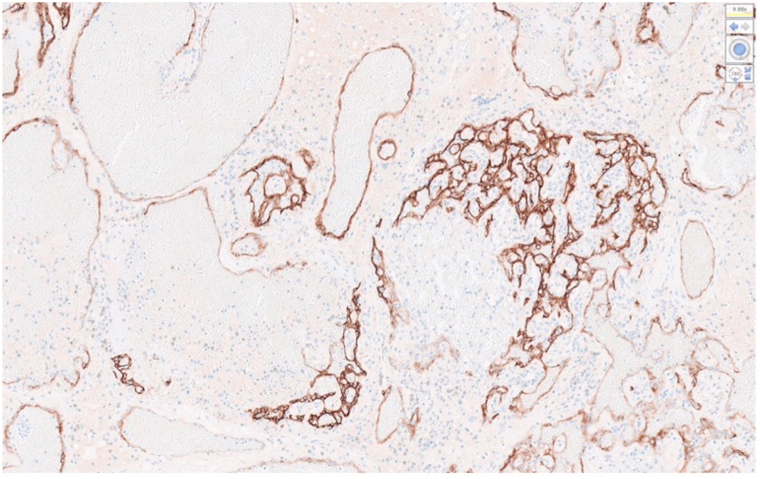
CD34 immunostaining of the excised specimen. Positive staining is restricted to vascular endothelial cells.

## Discussion

SCH is a benign vascular tumor that predominantly arises in the distal extremities; truncal involvement is extremely rare.^1^^,^^2^ It can occur across all age groups, with a peak in young adults (median age 32 years) and no sex predilection.^1^ Histopathologically, it is characterized by dilated vascular lumina surrounded by proliferating spindle cells, often with a multinodular architecture accompanied by intraluminal thrombus formation and organization.^3^ Immunohistochemically, vascular endothelial cells are positive for CD31 and CD34, whereas expression of these markers in the spindle cell component is limited.^3^ Reports of SCH arising outside the distal extremities are scarce; identified cases are summarized in Table I.

**Table I:** Published cases of SCH arising outside the extremities

Author (y)	Age/sex	Location	Size (cm)	Final Dx
Talan-Hranilović et al (2007)^4^	31/M	Spine (T1-T3)	5.3 × 1.2	SCH
Tosios et al (2008)^5^	29/F	Upper lip	1 × 0.7	SCH
Jena et al (2012)^2^	45/M	Abdominal wall	7 × 5	SCH
Present case	20s/M	Lower abdominal wall	7 × 4	SCH

*DFSP*, Dermatofibrosarcoma protuberans; *SCH*, spindle cell hemangioma.

DFSP is the most important differential diagnosis for spindle cell tumors of the trunk. DFSP demonstrates diffuse CD34 positivity throughout the tumor cells and grows infiltratively into the adipose tissue in a storiform pattern,^6^ whereas CD34 expression in SCH is predominantly restricted to vascular endothelial cells and infiltrative growth is absent—a critical distinction. Since the overall architecture of the tumor cannot be adequately assessed on biopsy specimens, comprehensive pathological evaluation of the excised specimen is essential. Other entities in the differential diagnosis include epithelioid hemangioendothelioma, epithelioid hemangioma, Kaposi sarcoma, and composite hemangioendothelioma, all of which can be distinguished from SCH by integrating morphological features, immunohistochemical profiles, and growth patterns. In this case, Kaposi sarcoma was excluded by HHV8 negativity and absent characteristic morphological features; spindle cell angiosarcoma was excluded by the low Ki-67 index and absent significant cytological atypia.

In the present case, 3 factors contributed to the initial misdiagnosis as DFSP on core needle biopsy. First, core needle biopsy did not allow adequate evaluation of the overall vascular architecture of the tumor, making it impossible to assess the storiform pattern and honeycomb-like adipose tissue infiltration characteristic of DFSP. Second, the abundant blood flow signals on ultrasound are not specific to DFSP and, in retrospect, should have been reinterpreted as suggestive of a vascular tumor. Third, CD34 positivity was misidentified as tumor-wide diffuse expression, directly leading to the misdiagnosis. When a CD34-positive hypervascular truncal tumor lacks the typical features of DFSP, vascular tumors including SCH should be included in the differential diagnosis, and comprehensive pathological evaluation of the excised specimen—with particular attention to the distribution pattern of CD34 positivity (diffuse throughout tumor cells vs restricted to vascular endothelial cells)—is essential for accurate diagnosis. In our case, the diagnostic pitfall was that CNB did not capture the characteristic vascular architecture of SCH. The limited biopsy specimen mainly showed spindle cell proliferation with CD34 positivity, leading to a preoperative diagnosis of DFSP. In retrospect, an incisional biopsy including both superficial and deeper components may have provided more informative tissue by allowing evaluation of the overall tumor architecture, vascular pattern, intraluminal thrombosis, adipose tissue infiltration, and the distribution of CD34 staining.

Treatment consists of surgical excision, which generally yields favorable outcomes.^1^ The local recurrence rate is relatively high (approximately 58%), but no metastases or tumor-related deaths have been reported, confirming the benign biological behavior of this tumor.^1^ In conclusion, we report a case of relatively large SCH arising in the trunk that was initially misdiagnosed as DFSP on core needle biopsy. Awareness of these diagnostic pitfalls and recognition of the limitations of biopsy material are essential when evaluating hypervascular spindle cell tumors of the trunk. Although IDH1/2 mutational analysis represents a valuable ancillary tool for confirming the diagnosis of SCH, it was not performed in the present case as a definitive diagnosis was established based on the morphological findings and immunohistochemical profile.

### Declaration of generative AI and AI-assisted technologies in the writing process

During the preparation of this work, the authors used ChatGPT and Claude for language editing and text structuring, and Elicit for literature search and organization. After using these tools, the authors reviewed and edited the content as needed and take full responsibility for the content of the published article.

## Conflicts of interest

None disclosed.
